# Positive Association Between Fluoroquinolone Exposure and Tendon Disorders: A Nationwide Population-Based Cohort Study in Taiwan

**DOI:** 10.3389/fphar.2022.814333

**Published:** 2022-03-21

**Authors:** Chun-Kai Chang, Wu-Chien Chien, Wan-Fu Hsu, Hao-Yu Chiao, Chi-Hsiang Chung, Yuan-Sheng Tzeng, Shao-Wei Huang, Kuang-Ling Ou, Chih-Chien Wang, Shyi-Jou Chen, Der-Shiun Wang

**Affiliations:** ^1^ Division of Plastic and Reconstructive Surgery, Department of Surgery, Tri-Service General Hospital, National Defense Medical Center, Taipei, Taiwan; ^2^ Department of Surgery, Zouying Branch of Kaohsiung Armed Forces General Hospital, Kaohsiung, Taiwan; ^3^ Department of Medical Research, Tri-Service General Hospital, National Defense Medical Center, Taipei, Taiwan; ^4^ School of Public Health, National Defense Medical Center, Taipei, Taiwan; ^5^ Graduate Institute of Life Sciences, National Defense Medical Center, Taipei, Taiwan; ^6^ Department of Pediatrics, Tri-Service General Hospital, School of Medicine, National Defense Medical Center, Taipei, Taiwan; ^7^ Taiwanese Injury Prevention and Safety Promotion Association, Taipei, Taiwan; ^8^ Graduate Institute of Clinical Medicine, College of Medicine, National Taiwan University, Taipei, Taiwan

**Keywords:** fluoroquinolone, tendinopathy, tendon rupture, statin, glucocorticoid, aromatase inhibitor

## Abstract

**Introduction:** Fluoroquinolone exposure is reportedly associated with a higher risk of tendon disorders, tendonitis, or tendon rupture. However, studies in East Asian populations have not confirmed these risks in patients with comorbidities or concomitant medication use. This cohort study was designed to investigate the associations among fluoroquinolone exposure, comorbidities, medication use, and tendon disorders in Taiwan.

**Materials and Methods:** This population-based, nationwide, observational, cohort study used data from the National Health Insurance Research database in Taiwan, a nationwide claims database that covers more than 99% of the Taiwanese population. The study period was from January 2000 to December 2015, and the median follow-up time was 11.05 ± 10.91 years. Patients who were exposed to fluoroquinolones for more than three consecutive days were enrolled, and patients without fluoroquinolone exposure who were matched by age, sex, and index year were enrolled as controls. The associations of comorbidities and concomitant medication use with tendon disorder occurrence were analyzed using Cox regression models.

**Results:** The incidence of tendon disorders were 6.61 and 3.34 per 10^5^ person-years in patients with and without fluoroquinolone exposure, respectively (adjusted hazard ratio, 1.423; 95% confidence interval [1.02,1.87]; *p* = 0.021). Sensitivity analyses yielded similar results. Patients under 18 and over 60 years with fluoroquinolone exposure; those with chronic kidney disease, diabetes, rheumatologic disease, cardiac disease, lipid disorder, or obesity; and those who concomitantly used statins, aromatase inhibitors, or glucocorticoids, had a significantly higher risk of tendon disorders.

**Conclusion:** The long-term risk of tendon disorders was higher in patients with fluoroquinolone exposure than in those without fluoroquinolone exposure. Clinicians should assess the benefits and risks of fluoroquinolone use in patients at high risk of tendon disorders who require fluoroquinolone administration.

## Introduction

Fluoroquinolones are effective and widely used antibiotics that have some advantages: they have good oral bioavailability, are widely distributed, and have broad-spectrum antimicrobial activity (Jameson, 2018). Although fluoroquinolones are used worldwide, antimicrobial resistance to this drug class is increasing (Robicsek et al., 2006). Furthermore, some serious adverse effects of fluoroquinolones have been reported e.g., *Clostridioides difficile* infection (Guh et al., 2017), hepatotoxicity (Orman et al., 2011), neuropathy (Morales D. et al., 2019), altered mental status, seizures (Sutter et al., 2015), pseudotumor cerebri (Sodhi et al., 2017), myasthenia gravis exacerbations (Sheikh et al., 2021), QT interval prolongation (Kang et al., 2001), aortic aneurysm and dissection (Lee et al., 2015), dysglycemia (Chou et al., 2013), retinal detachment (Etminan et al., 2012), tendinopathy, and other tendon disorders (Berger et al., 2017). Thus, fluoroquinolone use needs to be carefully considered for patients in whom the benefits outweigh the risks.

Tendinopathy and tendon rupture are severe problems resulting from daily exercise, even when standing or walking. The Achilles tendon is the largest tendon in the body. It endures strain and risks rupture from running, jumping, sudden acceleration or deceleration, overuse, vascular disease, neuropathy, and rheumatologic diseases, which may cause tendon degeneration. The hallmark Achilles tendon problems appear to result from tendon damage, weakness, and inelasticity (von Rickenbach et al., 2021). The incidence of Achilles tendon rupture in the general population is approximately 5–10 per 100,000 (Sheth et al., 2017), although it may be higher in different regions and populations; the overall incidence is increasing (Lantto et al., 2014). Limited evidence has revealed that cold-weather training, foot misalignment, poor running mechanics, inappropriate footwear, weakness in plantar flexion, age, male sex, obesity, and leg-length discrepancy may be associated with an increased risk of Achilles tendinopathy or other problems, e.g., calcifying tendinitis of the shoulder and patellar tendinitis, among others (van der Vlist et al., 2019). Some systemic diseases, including cardiac disease, diabetes mellitus (DM), obesity, lipid disorders, and rheumatologic disorders have been reported in patients with tendon disorders (van der Vlist et al., 2019). Moreover, tendon disorders are reportedly associated with the use of statins (Marie et al., 2008), aromatase inhibitors (Laroche et al., 2017; Mitsimponas et al., 2018), and glucocorticoids (Wise et al., 2012).

The use of fluoroquinolones is associated with rare cases of Achilles tendinopathy or tendon rupture. Zabraniecki et al. (1996) reported six cases of fluoroquinolone-induced tendinopathy. The risk of tendon disorders was higher in Japanese patients who received fluoroquinolones than those who received cephalosporins (Hori et al., 2012). Moreover, a population-based case-control study in the United Kingdom reported that exposure to fluoroquinolones increased the risk of Achilles tendon rupture and that this risk was highest among elderly patients who were concomitantly treated with corticosteroids (van der Linden et al., 2003). In contrast, patients who used fluoroquinolones did not have a relatively higher risk of musculoskeletal adverse events, Achilles’ tendon rupture, or collagen-associated adverse effects (Yu et al., 2020; Wang et al., 2020; Chinen et al., 2021). A study of 46,776 patients treated with fluoroquinolones found 3.2 cases of tendon problems for every 1,000 years of exposure (van der Linden et al., 2002). According to a large case-control study, the incidence rate of tendon rupture associated with fluoroquinolone therapy is estimated to be 12 per 100,000 treatment episodes (Corrao et al., 2006). However, most of the studies were case-control studies, and only two large-scale population-based cohort studies were conducted; in Denmark (Sode et al., 2007) and in the United Kingdom (Persson and Jick, 2019). Both studies confirmed fluoroquinolone exposure to be associated with an increased risk of Achilles tendon rupture; however, the incidence was low. Furthermore, the risk of Achilles tendon rupture increases with concomitant use of corticosteroids. Therefore, in this study, we aimed to investigate the associations of fluoroquinolone exposure, comorbidities, and concomitant medication use with the risk of tendon disorder development in Taiwan.

## Materials and Methods

### Data Source

We conducted this retrospective, cohort study using data from the National Health Insurance Research database (NHIRD) in Taiwan (Hsieh et al., 2019). The original database includes the medical information of all persons, i.e., nearly 99% of the 23 million residents of Taiwan, who were covered by the National Health Insurance Program (NHIP) established in 1995. As of June 2009, more than 97% of medical service providers in Taiwan participated in the NHIP (i.e., approximately 23 million beneficiaries, or more than 99% of the entire population of Taiwan). The NHIRD uses the International Classification of Diseases, 9th and 10th revisions, Clinical Modification (ICD-9-CM and ICD-10) codes to record diagnoses.

Every agent has a unique code in the NHIRD. A total of seven categories (ofloxacin, levofloxacin, ciprofloxacin, norfloxacin, pefloxacin, gemifloxacin, and moxifloxacin) and 127 different fluoroquinolone agents (e.g., Cravit and Avelox, among others) have been registered in the NHIRD system. Furthermore, 593 agents, including statins, aromatase inhibitors, and corticosteroids, were also analyzed in this study. Licensed medical record technicians review and verify the diagnostic codes before a claim for insurance payment to the hospital is approved. Moreover, the NHIP administration randomly reviews outpatient visit records, and periodically reviews inpatient claims to verify the accuracy of diagnoses. Therefore, the NHIRD data are considered reliable. Consequently, we used the NHIRD data in this study to examine the association between fluoroquinolone use and tendon disorder occurrence.

### Study Design and Participants

In this retrospective, matched, cohort study, patients who were exposed to fluoroquinolones for more than 3 days between 1 January 2000 and 31 December 2015 were included in the fluoroquinolone group. Day one of continuous fluoroquinolone exposure of more than 3 days was assigned as the index date. Patients without fluoroquinolone exposure or with less than 3 days of fluoroquinolone exposure throughout the study period were age-, sex-, and index year-matched (1:1) to those in the fluoroquinolone group and assigned to the control group. Individuals with fluoroquinolone exposure before the index date, those with tendon disorders before tracking or without tracking, as well as those of unknown age or sex, were excluded from the analysis.

### Covariates and Comorbidities

We considered the covariates of sex, age, season, geographical residence area (northern, central, southern, or eastern Taiwan), residence area urbanization level (levels 1–4), type of hospital (hospital center, regional hospital, or local hospital), and insurance premium category (in New Taiwan Dollars; <18,000, 18,000–34,999, or ≥35,000). Urbanization level was defined according to population size and various indicators of development. Urbanization level 1 was defined as an area with >1,250,000 inhabitants and a specific political, economic, cultural, and metropolitan development designation. Urbanization level 2 was defined as an area with 500,000 to 1,249,999 inhabitants and an important role in the political system, economy, and culture. Urbanization levels 3 and 4 were defined as areas with 149,999–499,999 and <149,999 inhabitants, respectively.

The comorbidities considered were chronic kidney disease (CKD), DM, obesity, rheumatologic disease, cardiac disease, and lipid disorders when the patient was first indexed. Moreover, we evaluated the use of drugs such as statins, aromatase inhibitors, and glucocorticoids. Outpatient department (OPD) and emergency department (ED) visits, inpatient department (IPD) stays, and intensive care unit (ICU) stays were also investigated. All comorbidities and the use of other drugs (continuous use for more than 3 days) were identified when the cases or controls were assigned and tendon disorders were coded within 30 days or at the end of the study period. Further, we analyzed the duration of use of different fluoroquinolone agents.

### Outcomes

Participants were followed up from the index date until the onset of a tendon disorder, withdrawal from the NHIP, or the end of 2015. The tendon disorders considered were Achilles tendon rupture, Achilles tendonitis, and other tendon diseases.

The ICD-9 and 10 CM codes used to classify Achilles tendon rupture, tendonitis, other tendon diseases, and comorbidities, as well as the specific drug codes, are listed in Supplementary Table S1.

### Statistical Analyses

All analyses were performed using SPSS (version 22, SPSS Inc., Chicago, IL, United States). The Chi-square test and Student’s *t*-test were used to evaluate categorical and continuous variable distributions, respectively. Fisher’s exact test was used to examine the differences between the two cohorts in terms of categorical variables. We used the competing risk analysis approach put forward by Fine and Gray to identify the risk of tendon disorders (competing with death) in patients with fluoroquinolone exposure (Marie et al., 2008; van der Vlist et al., 2019). A sensitivity analysis, excluding the diagnosis of tendon disorders, within the first year and the first 5 years, was conducted to avoid protopathic bias. The differences in risk between the fluoroquinolone and control cohorts were estimated using Kaplan-Meier analysis with the log-rank test. A two-tailed *p*-value < 0.05 was considered statistically significant.

### Ethics

The Institutional Review Board of the Tri-Service General Hospital approved this study (TSGHIRB: B-110-43). This study was conducted strictly according to the Declaration of Helsinki. This is a secondary data analysis article from the NHIRD and all necessary permissions were obtained to access and use the data. Informed consent from the participants was waived because the NHIRD contains de-identified information, which does not affect the rights and welfare of the participants.

## Results

### Patient Characteristics

**FIGURE 1:**
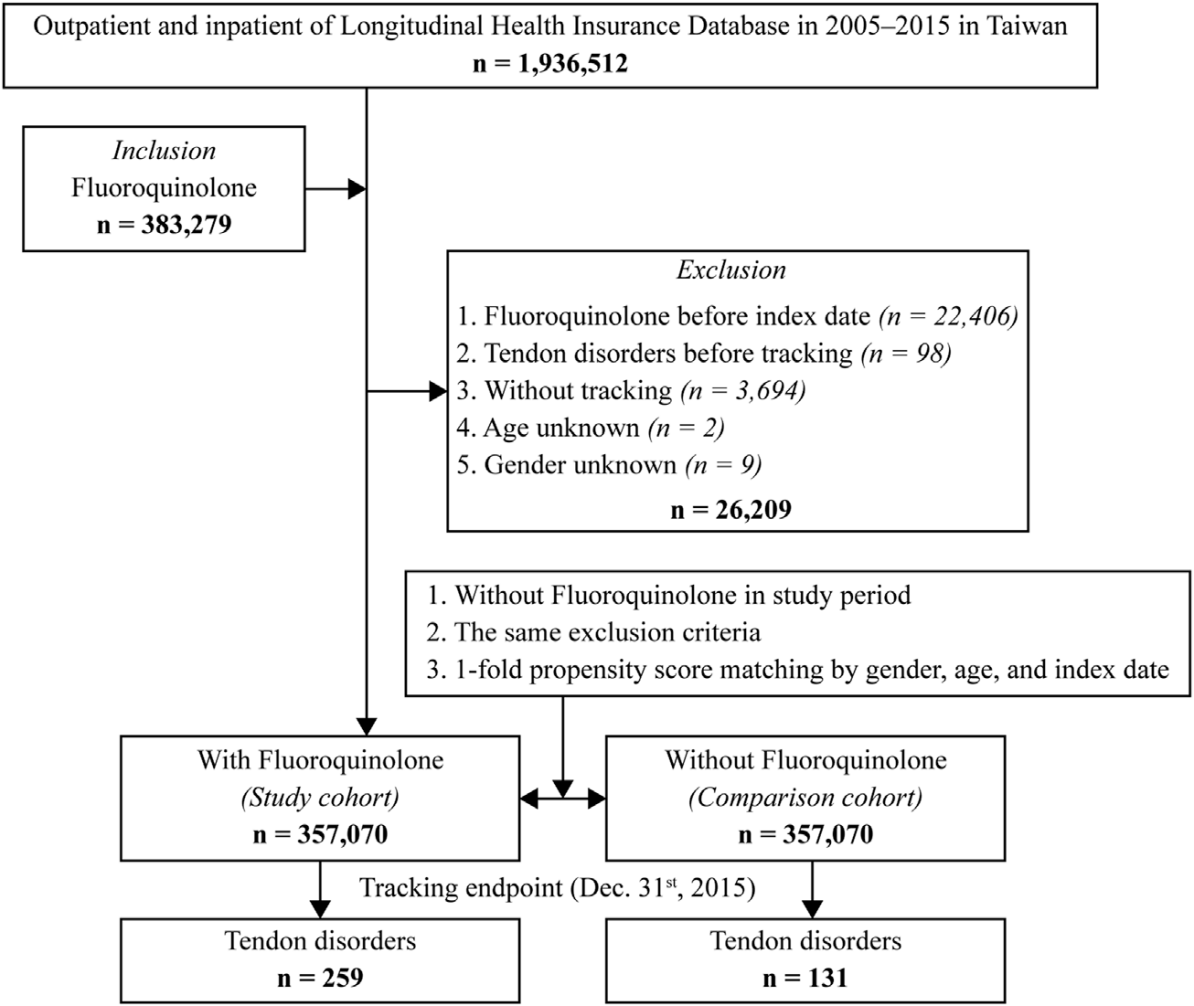
Flowchart of the study sample selection.

A total of 1,936,512 individuals (approximately 10% of the Taiwanese population) were identified in the database between January 2000 and December 2015, of whom 3,83,279 patients received fluoroquinolones for more than 3 days. We excluded 26,209 patients based on the exclusion criteria; hence, 3,57,070 patients were enrolled in the study. Furthermore, we assigned 3,57,070 individuals without fluoroquinolone exposure to a control group which was matched by sex, age, and index date (Figure 1). The mean overall follow-up duration was 11.05 ± 10.91 years, and the mean follow-up time to tendon disorder occurrence was 7.52 ± 7.28 years (Supplementary Tables S2-1 and S2-2). The mean age was 45.99 ± 18.92 years and 51.6% of the individuals were male. The fluoroquinolone exposure group had a significantly higher proportion of the following covariates: CKD (*p* < 0.001, Table 1), DM (*p* < 0.001, Table 1), rheumatologic disease (*p* < 0.001, Table 1), cardiac disease (*p* < 0.001, Table 1), statin use (*p* = 0.01, Table 1), and glucocorticoid use (*p* < 0.001, Table 1). However, the differences in the covariates between the exposure and control groups were less than 2%, and most of the differences were within 1%.

**TABLE 1:** Baseline characteristics.

Variables	Total		Fluoroquinolone group		Control group		*p*
	N	%	n	%	n	%	
Total	714,140		357,070	50.00	357,070	50.00	
Sex							0.999
Male	368,262	51.57	184,131	51.57	184,131	51.57	
Female	345,878	48.43	172,939	48.43	172,939	48.43	
Age (yrs)	45.99 ± 18.92		45.96 ± 18.72		46.02 ± 19.11		0.180
Age groups (yrs)							0.999
0–17	146,242	20.48	73,121	20.48	73,121	20.48	
18–34	194,530	27.24	97,265	27.24	97,265	27.24	
35–59	206,934	28.98	103,467	28.98	103,467	28.98	
≥ 60	166,434	23.31	83,217	23.31	83,217	23.31	
Insurance premium (NT$)							**<0.001**
<18,000	595,133	83.34	297,121	83.21	298,012	83.46	
18,000–34,999	75,219	10.53	37,608	10.53	37,611	10.53	
≥ 35,000	43,788	6.13	22,341	6.26	21,447	6.01	
CKD							**<0.001**
Without	649,874	91.00	323,969	90.73	325,905	91.27	
With	64,266	9.00	33,101	9.27	31,165	8.73	
DM							**<0.001**
Without	614,006	85.98	303,925	85.12	310,081	86.84	
With	100,134	14.02	53,145	14.88	46,989	13.16	
Obesity							0.779
Without	713,937	99.97	356,966	99.97	356,971	99.97	
With	203	0.03	104	0.03	99	0.03	
Rheumatologic disease							**<0.001**
Without	689,810	96.59	344,619	96.51	345,191	96.67	
With	24,330	3.41	12,451	3.49	11,879	3.33	
Cardiac disease							**<0.001**
Without	647,498	90.67	321,642	90.08	325,856	91.26	
With	66,642	9.33	35,428	9.92	31,214	8.74	
Lipid disorders							0.078
Without	707,134	99.02	353,493	99.00	353,641	99.04	
With	7,006	0.98	3,577	1.00	3,429	0.96	
Statins							**0.010**
Without	706,767	98.97	353,274	98.94	353,493	99.00	
With	7,373	1.03	3,796	1.06	3,577	1.00	
Aromatase inhibitors							0.101
Without	711,817	99.67	355,869	99.66	355,948	99.69	
With	2,323	0.33	1,201	0.34	1,122	0.31	
Glucocorticoids							**<0.001**
Without	711,263	99.60	355,497	99.56	355,766	99.63	
With	2,877	0.40	1,573	0.44	1,304	0.37	
OPD/ED visits							**0.002**
1–2	77,116	10.80	38,971	10.91	38,145	10.68	
≥ 3	637,024	89.20	318,099	89.09	318,925	89.32	
IPD stays (days)							**0.006**
0	428,279	59.97	213,478	59.79	214,801	60.16	
1–2	110,717	15.50	55,579	15.57	55,138	15.44	
≥ 3	175,144	24.53	88,013	24.65	87,131	24.40	
ICU days							**<0.001**
0	538,225	75.37	268,452	75.18	269,773	75.55	
1–6	141,527	19.82	70,505	19.75	71,022	19.89	
≥ 7	34,388	4.82	18,113	5.07	16,275	4.56	
Season							0.999
Spring (Mar–May)	178,130	24.94	89,065	24.94	89,065	24.94	
Summer (Jun–Aug)	180,248	25.24	90,124	25.24	90,124	25.24	
Autumn (Sep–Nov)	178,262	24.96	89,131	24.96	89,131	24.96	
Winter (Dec–Feb)	177,500	24.86	88,750	24.86	88,750	24.86	
Location							**0.003**
Northern Taiwan	201,813	28.26	101,251	28.36	100,562	28.16	
Middle Taiwan	197,466	27.65	98,453	27.57	99,013	27.73	
Southern Taiwan	199,647	27.96	99,862	27.97	99,785	27.95	
Eastern Taiwan	85,938	12.03	42,641	11.94	43,297	12.13	
Outlets islands	29,276	4.10	14,863	4.16	14,413	4.04	
Urbanization level							**0.323**
1 (Highest)	194,390	27.22	97,256	27.24	97,134	27.20	
2	231,307	32.39	115,297	32.29	116,010	32.49	
3	114,013	15.97	57,121	16.00	56,892	15.93	
4 (Lowest)	174,430	24.43	87,396	24.48	87,034	24.37	
Level of care							**0.018**
Hospital center	242,853	34.01	121,875	34.13	120,978	33.88	
Regional hospital	247,896	34.71	124,010	34.73	123,886	34.70	
Local hospital	223,391	31.28	111,185	31.14	112,206	31.42	

p values were evaluated using the Chi-square test/Fisher’s exact test and the Student’s *t*-test for categorical and continuous variables, respectively.

CKD, chronic kidney disease; DM, diabetes mellitus; ED, emergency department; IPD, inpatient department; OPD, outpatient department; ICU, intensive care unit

### Participant Characteristics at the Study Endpoint

**FIGURE 2:**
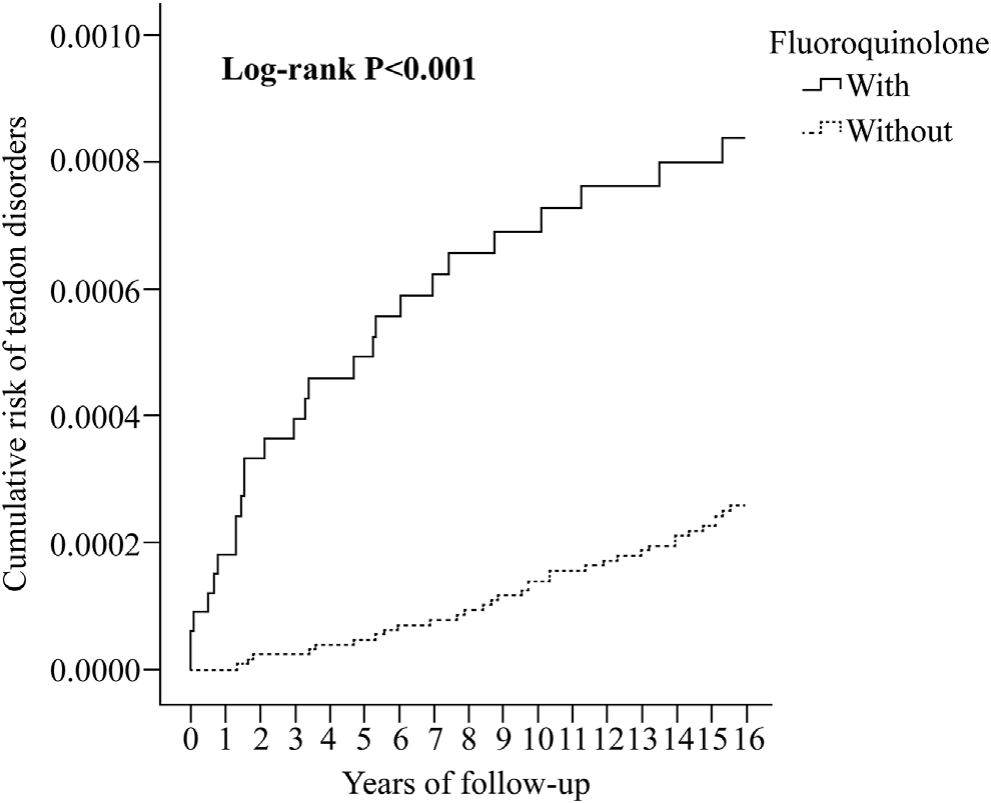
Kaplan-Meier analysis of the cumulative risk of tendon disorders stratified by fluoroquinolone exposure using the log-rank test.

At the study endpoint, 259 (0.07%) and 131 (0.04%) individuals in the fluoroquinolone and control groups developed tendon disorders, respectively (*p* < 0.001, Supplementary Table S3). The incidence was 6.61 and 3.34 per 10^5^ person-years (PYs) in the fluoroquinolone and control groups, respectively (adjusted hazard ratio [aHR] 1.45; 95% confidence interval [CI] [1.04, 1.90]; *p* = 0.011) (Supplementary Table S4). Kaplan-Meier analysis revealed that the 15-years cumulative incidence of tendon disorders was significantly higher in the fluoroquinolone cohort (*p* < 0.001; Figure 2).

### Factors Associated With Tendon Disorders

In the Cox regression analysis (Table 2), the crude HR for tendon disorders in the fluoroquinolone group was 1.51 (95% CI [1.11, 1.97]; *p* < 0.001). After adjusting for sex, age, insurance premium, comorbidities, drug use, ED/OPD visits, IPD/ICU stay, season, level of urbanization, and level of care, patients in the fluoroquinolone exposure group showed an increased risk of developing tendon disorders, with an aHR of 1.42 (95% CI [1.02, 1.87]; *p =* 0.021). Kaplan-Meier analysis also showed a higher cumulative risk of tendon disorders in the fluoroquinolone group (*p* < 0.001) (Figure 2). In this cohort, individuals with CKD (*p* = 0.028), DM (*p* < 0.001), rheumatologic disease (*p* = 0.046), and cardiac disease (*p* < 0.001) were more predisposed to tendon disorder development than those with obesity (*p* = 0.559) and lipid disorders (*p* = 0.117). Individuals who used statins had a slightly increased risk of tendon disorders, with a crude HR of 1.41 (95% CI [1.12, 1.86]; *p* < 0.001). Although the impact of statin use was not statistically significant after adjusting for covariates, it trended toward significance (95% CI [0.998, 1.68]; *p* = 0.053). The use of aromatase inhibitors (*p* = 0.423) or glucocorticoids (*p* = 0.470) had no significant effect on the development of tendon disorders. Individuals living in regions with higher urbanization levels (*p* < 0.001) showed an increased risk of tendon disorders; moreover, most patients with tendon disorders were treated in hospital centers (*p* < 0.001) and regional hospitals (*p* < 0.033).

**TABLE 2:** Risk factors of tendon disorders using Cox regression analysis.

Variables	Crude HR	95% CI	95% CI	*p*	Adjusted HR	95% CI	95% CI	*p*
Fluoroquinolone								
Without	Reference				Reference			
With	1.513	1.110	1.972	**<0.001**	1.423	1.023	1.869	**0.021**
Sex								
Male	1.386	0.861	1.572	0.174	1.299	0.813	1.520	0.189
Female	Reference				Reference			
Age groups (yrs)								
0–17	Reference				Reference			
18–34	0.986	0.797	1.185	0.235	0.973	0.724	1.048	0.277
35–59	0.913	0.786	1.104	0.372	0.842	0.625	1.001	0.389
≥ 60	1.026	0.834	1.264	0.196	1.003	0.798	1.125	0.201
Insurance premium (NT$)								
<18,000	Reference				Reference			
18,000–34,999	0.985	0.822	1.253	0.144	0.954	0.798	1.186	0.201
≥ 35,000	0.971	0.803	1.126	0.189	0.901	0.765	1.074	0.235
CKD								
Without	Reference				Reference			
With	1.284	1.050	1.371	0.001	1.189	1.022	1.298	0.028
DM								
Without	Reference				Reference			
With	1.598	1.204	1.893	**<0.001**	1.486	1.135	1.776	**<0.001**
Obesity								
Without	Reference				Reference			
With	1.865	0.565	3.795	0.502	1.533	0.374	2.863	0.559
Rheumatologic disease								
Without	Reference				Reference			
With	1.241	1.095	1.386	**<0.001**	1.151	1.003	1.278	0.046
Cardiac disease								
Without	Reference				Reference			
With	1.732	1.302	2.203	**<0.001**	1.560	1.128	1.999	**<0.001**
Lipid disorders								
Without	Reference				Reference			
With	1.342	1.086	1.785	**<0.001**	1.188	0.843	1.590	0.117
Statins								
Without	Reference				Reference			
With	1.405	1.124	1.862	**<0.001**	1.204	0.998	1.684	0.053
Aromatase inhibitors								
Without	Reference				Reference			
With	1.382	0.688	2.141	0.379	1.264	0.597	2.010	0.423
Glucocorticoids								
Without	Reference				Reference			
With	1.335	0.624	2.005	0.386	1.227	0.531	1.988	0.470
OPD/ED visits								
1–2	Reference				Reference			
≥ 3	1.227	1.049	1.426	**0.001**	1.215	1.038	1.409	**0.013**
IPD stays								
0	Reference				Reference			
1–2	1.566	1.209	2.785	**<0.001**	1.302	1.050	2.441	**0.001**
≥ 3	2.874	1.488	3.862	**<0.001**	1.993	1.287	2.658	**<0.001**
ICU days								
0	Reference				Reference			
1–6	2.065	1.488	2.978	**<0.001**	1.888	1.267	2.706	**<0.001**
≥ 7	2.413	1.892	3.304	**<0.001**	2.245	1.683	3.025	**<0.001**
Season								
Spring	Reference				Reference			
Summer	1.098	0.562	1.578	0.426	1.035	0.513	1.482	0.499
Autumn	1.176	0.671	1.692	0.379	1.099	0.600	1.570	0.403
Winter	1.199	0.688	1.707	0.311	1.122	0.611	1.664	0.382
Location					**Multicollinearity with urbanization level**			
Northern Taiwan	Reference				**Multicollinearity with urbanization level**			
Middle Taiwan	0.986	0.777	1.230	0.311	**Multicollinearity with urbanization level**			
Southern Taiwan	0.873	0.725	1.184	0.397	**Multicollinearity with urbanization level**			
Eastern Taiwan	0.586	0.301	0.984	**0.017**	**Multicollinearity with urbanization level**			
Outlets islands	0.678	0.005	4.821	0.876	**Multicollinearity with urbanization level**			
Urbanization level								
1 (The highest)	1.335	1.127	1.703	**<0.001**	1.324	1.089	1.589	**<0.001**
2	1.312	1.104	1.670	**<0.001**	1.299	1.056	1.572	**<0.001**
3	1.196	0.975	1.456	0.079	1.027	0.864	1.344	0.134
4 (The lowest)	Reference				Reference			
Level of care								
Hospital center	2.446	1.708	3.011	**<0.001**	1.882	1.501	2.279	**<0.001**
Regional hospital	1.495	1.232	1.680	**<0.001**	1.265	1.013	1.489	**0.033**
Local hospital	Reference				Reference			

**Adjusted HR:** Adjusted variables are listed in the table.CKD, chronic kidney disease; DM, diabetes mellitus; ED, emergency department; IPD, inpatient department; OPD, outpatient department; ICU, intensive care unit; HR, hazard ratio; CI, confidence interval

### Risk Factors of Tendon Disorders Stratified Using Cox Regression

Fluoroquinolone-exposed individuals had a higher risk of tendon disorders than those without fluoroquinolone exposure (aHR 1.423; 95% CI [1.02, 1.87]; *p* = 0.021) (Table 3). Moreover, fluoroquinolone-exposed individuals under 18 and over 60 years were more likely to develop tendon disorders than those without fluoroquinolone exposure (aHR 1.58 and 1.59, respectively). In addition, patients with fluroquinolone exposure with CKD, DM, obesity, rheumatologic disease, cardiac disease, and lipid disorders (adjusted HR: 1.50, 1.43, 1.74, 1.58, 1.64, 2.00, *p* = 0.001, 0.015, <0.001, <0.001, <0.001, <0.001) had a slightly higher risk of tendon disorders, compared to those without those chronic diseases. The patients with fluroquinolone exposure who were also using statins, aromatase inhibitors, or glucocorticoids (adjusted HR: 1.91, 1.43, 1.51, respectively, *p* < 0.001) had a higher risk of tendon disorders, compared to those without exposure to those medications. Patients with fluroquinolone exposure who visited the OPD/ER more than three times (adjusted HR: 1.43, *p* = 0.019), had a higher risk of tendon disorders than patients who visited the OPD/ER 1-2 times. Patients with fluroquinolone exposure with more than 3-days IPD stays, 1–2-days IPD stays, ≧7 days ICU stay, 1–6 days ICU stay (adjusted HR:1.57, 1.51, 1.45, 1.39, *p* < 0.001, <0.001, 0.006, 0.049, respectively) showed a higher risk of tendon disorders compared to those without in-hospital or ICU stays. The patients who lived in level 1 and 2 urbanization areas (adjusted HR: 1.59, 1.40, *p* < 0.001, 0.045, respectively) had a higher risk of tendon disorders, compared to those who lived in a level 4 urbanization area. The patients who sought medical help in hospital centers (adjusted HR: 1.843 *p* < 0.001) had a higher risk of tendon disorders compared to those who went to local hospitals.

**TABLE 3:** Factors of tendon disorders stratified by variables listed in the table by using Cox regression.

Fluoroquinolone	With			Without (*Reference*)			With *vs.* Without (*Reference*)			
Stratified	Events	PYs	Rate (per 10^5^ PYs)	Events	PYs	Rate (per 10^5^ PYs)	Adjusted HR	95% CI	95% CI	*p*
Overall	259	3,919,057.93	6.61	131	3,920,618.42	3.34	1.423	1.023	1.869	**0.021**
Sex										
Male	135	2,019,927.07	6.68	69	2,047,535.72	3.37	1.427	1.026	1.874	**0.019**
Female	124	1,899,130.86	6.53	62	1,873,082.70	3.31	1.419	1.020	1.864	**0.023**
Age group (yrs)										
0-17	44	794,787.43	5.54	20	790,910.26	2.53	1.575	1.132	2.069	**<0.001**
18–34	72	1,055,981.65	6.82	39	1,068,973.84	3.65	1.345	0.967	1.766	0.189
35–59	82	1,134,660.08	7.23	45	1,149,073.11	3.92	1.328	0.954	1.744	0.131
≧60	61	933,628.77	6.53	27	911,661.21	2.96	1.587	1.141	2.085	**<0.001**
Insurance premium (NT$)										
<18,000	227	3,259,416.24	6.96	115	3,313,892.98	3.47	1.444	1.038	1.896	**0.017**
18,000–34,999	21	412,559.95	5.09	11	418,234.12	2.63	1.392	1.001	1.829	**0.049**
≧35,000	11	247,081.74	4.45	5	188,491.32	2.65	1.207	0.868	1.586	0.246
CKD										
Without	187	3,555,945.08	5.26	98	3,574,063.59	2.74	1.380	0.992	1.812	0.059
With	72	363,112.85	19.83	33	346,554.83	9.52	1.498	1.077	1.968	**0.001**
DM										
Without	163	3,336,056.76	4.89	87	3,398,100.79	2.56	1.373	0.987	1.803	0.063
With	96	583,001.17	16.47	44	522,517.63	8.42	1.429	1.035	1.897	**0.015**
Obesity										
Without	254	3,917,921.38	6.48	129	3,919,519.75	3.29	1.417	1.019	1.861	**0.038**
With	5	1,136.55	439.93	2	1,098.67	182.04	1.739	1.250	2.284	**<0.001**
Rheumatologic disease										
Without	234	3,782,469.07	6.19	120	3,788,522.70	3.17	1.405	1.010	1.846	**0.040**
With	25	136,588.86	18.30	11	132,095.72	8.33	1.581	1.137	2.077	**<0.001**
Cardiac disease										
Without	213	3,530,411.64	6.03	113	3,573,518.17	3.16	1.373	0.987	1.803	0.062
With	46	388,646.29	11.84	18	347,100.25	5.19	1.642	1.180	2.157	**<0.001**
Lipid disorders										
Without	239	3,879,817.68	6.16	124	3,882,486.21	3.19	1.388	0.998	1.823	0.051
With	20	39,240.25	50.97	7	38,132.21	18.36	1.998	1.436	2.624	**<0.001**
Statins										
Without	234	3,877,415.82	6.03	122	3,880,842.20	3.14	1.381	0.993	1.814	0.059
With	25	41,642.11	60.04	9	39,776.22	22.63	1.909	1.372	2.507	**<0.001**
Aromatase inhibitors										
Without	238	3,905,882.64	6.09	121	3,908,141.32	3.10	1.416	1.018	1.860	**0.034**
With	21	13,175.29	159.39	10	12,477.10	80.15	1.431	1.029	1.879	**0.020**
Glucocorticoids										
Without	239	3,901,802.07	6.13	123	3,906,117.43	3.15	1.400	1.006	1.838	**0.045**
With	20	17,255.86	115.90	8	14,500.99	55.17	1.511	1.087	1.985	**0.001**
OPD/ER visits										
1–2	43	427,512.89	10.06	22	424,174.83	5.19	1.395	1.003	1.833	**0.047**
≧3	216	3,491,545.04	6.19	109	3,496,443.59	3.12	1.428	1.026	1.875	**0.019**
IPD stays										
0	144	2,343,049.27	6.15	79	2,388,588.29	3.31	1.337	0.961	1.756	0.086
1–2	46	609,710.25	7.54	22	613,135.72	3.59	1.513	1.088	1.987	**<0.001**
≧3	69	966,298.41	7.14	30	918,894.41	3.26	1.574	1.131	2.067	**<0.001**
ICU days										
0	155	2,944,917.86	5.26	90	2,999,874.82	3.00	1.262	0.907	1.658	0.177
1–6	36	773,439.11	4.65	19	789,765.71	2.41	1.392	1.001	1.828	**0.049**
≧7	68	200,700.96	33.88	22	130,977.89	16.80	1.451	1.043	1.906	**0.006**
Season										
Spring	59	976,450.83	6.04	32	991,070.83	3.23	1.346	0.968	1.768	0.115
Summer	71	988,737.12	7.18	38	1,003,881.75	3.79	1.365	0.981	1.793	0.060
Autumn	63	977,932.05	6.44	31	992,970.66	3.12	1.485	1.067	1.950	**0.009**
Winter	66	975,937.93	6.76	30	932,695.18	3.22	1.513	1.087	1.987	**0.001**
Urbanization level										
1 (The highest)	72	1,067,435.33	6.75	33	1,082,376.33	3.05	1.592	1.144	2.091	**<0.001**
2	89	1,242,879.86	7.16	47	1,276,420.46	3.68	1.399	1.006	1.838	**0.045**
3	22	614,013.07	3.58	11	590,704.71	1.86	1.384	0.995	1.818	0.055
4 (The lowest)	76	994,729.67	7.64	40	971,116.92	4.12	1.335	0.959	1.753	0.128
Level of care										
Hospital center	84	1,338,032.66	6.28	33	1,346,897.59	2.45	1.843	1.325	2.421	**<0.001**
Regional hospital	92	1,369,780.05	6.72	51	1,378,981.31	3.70	1.307	0.939	1.716	0.177
Local hospital	83	1,211,245.22	6.85	47	1,194,739.52	3.93	1.253	0.901	1.646	0.203

PYs, Person-years; Adjusted HR, Adjusted hazard ratio: Adjusted for the variables listed in Table 3; CI, confidence interval.

The overall incidence of tendon disorder in patients with and without fluoroquinolone exposure was 6.61 and 3.34 per 10^5^ PYs, respectively. The incidence of tendon disorder was higher among fluoroquinolone-exposed patients with CKD, DM, obesity, rheumatologic disease, cardiac disease, and lipid disorders (19.83, 16.47, 439.93, 18.30, 11.84, and 50.97 per 10^5^ PYs, respectively) than among patients without fluoroquinolone exposure who had these comorbidities. In particular, fluoroquinolone-exposed patients who were obese had the highest proportion of tendon disorders (aHR 1.74; 95% CI [1.25, 2.28]; *p* < 0.001). We also analyzed the effects of exposure to statins, aromatase inhibitors, and glucocorticoids on the occurrence of tendon disorders and found that only patients who used statins were predisposed to developing tendon disorders. However, the significance of statin use was lost after adjusting for other covariates. After stratifying by statin, aromatase inhibitor, and glucocorticoid use, we found that the incidence of tendon disorders in the fluoroquinolone group was 60.04, 159.39, and 115.90 per 10^5^ PYs, respectively.

### Risk Factors of Tendon Disorders Among Different Cox Regression Models

As shown in Supplementary Table S5, different models were designed for analyzing the differences between patient sources (OPD/ER only and IPD/ICU), fluoroquinolone agents (ofloxacin, levofloxacin, ciprofloxacin, norfloxacin, pefloxacin, gemifloxacin, and moxifloxacin), and days of fluoroquinolone exposure (≤14 days/15–30 days/>30 days).

Patients who were treated in the hospital, either in a normal ward or in the ICU, were considered to have more severe infections than those who were only treated in the OPD or ED. We analyzed the patients treated in the hospital and ICU together as a subgroup and classified those who received care from the OPD/ED together as the other subgroup and then analyzed the risk of tendon disorders. In model 1, the aHR for patients from the IPD or ICU was 1.57 (95% CI [1.13, 2.07]; *p* < 0.001), which was significantly higher than that of patients who only visited the OPD/ED. In model 2, we analyzed the relationship between different agents of fluoroquinolones. After adjusting for the seven fluoroquinolone agents, levofloxacin, norfloxacin, and pefloxacin exposure were associated with a higher risk of developing tendon disorders. In model 3, individuals who used fluoroquinolones for more than 30 days had a significantly higher risk of developing a tendon disorder (aHR 1.792; 95% CI [(1.29, 2.36]; *p* < 0.001).

### Sensitivity Test for Risk Factors of Tendon Disorders, Using Cox Regression Analysis, With and Without the Fine–Gray Competing Risk Model

After excluding all participants enrolled in the first year and the first 5 years of the study, the risk factors (including fluoroquinolone exposure) for tendon disorders remained significantly high. Overall, the aHR was 1.45 (95% CI [1.04, 1.90]; *p =* 0.011); the aHR after excluding participants enrolled in the first year was 1.40 (95% CI [1.01, 1.83]; *p =* 0.038), and the aHR after excluding participants enrolled in the first 5 years was 1.43 (95% CI [1.02, 1.87]; *p =* 0.022) (Supplementary Table S4).

## Discussion

In this retrospective study, we found that fluoroquinolone exposure had a long-term effect of higher risk of tendon disorders. Moreover, individuals who used fluoroquinolones for more than 30 days with more severe disease (indicated by the presence of CKD, DM, rheumatologic or cardiac disease, as well as more OPD/ED visits or IPD stays, or a long ICU stay) had a higher risk of tendon disorders.

In this analysis, patients were considered exposed to fluoroquinolones throughout the follow-up period. This design is different from the Danish study with post-exposure for 90 days (Sode et al., 2007) or the British study on post-exposure of less than 30, 31–60, or 61–180 days (Persson and Jick, 2019). In line with Persson and Jick’s outcome, the fluoroquinolone attributable risk of Achilles tendon rupture was low. The fluoroquinolone exposure may not cause tendon rupture or tendinopathy directly unless there are other existing risk factors, such as corticosteroid use or DM.

Our study is the first to report that fluoroquinolone exposure predisposes children to tendon disorders. Specifically, in the current study, fluoroquinolone exposure was found to significantly increase the risk of tendon disorders in both men and women, as well as in people younger than 18 and older than 60 years. A previous study revealed that there was no difference in collagen-associated disorders in children with or without fluoroquinolone exposure (Yu et al., 2020). However, compared with the previous study, the present study had different enrollment criteria and a different follow-up period. Specifically, the previous study enrolled children with fluoroquinolone exposure for more than 5 days and followed-up the children for 6 months. In contrast, this study enrolled patients with fluoroquinolone exposure for more than 3 days and the mean follow-up period was 7.52 ± 7.28 years (Supplementary Tables S2-1 and S2-2). In the future, the effects of fluoroquinolone exposure on tendon disorders will need to be examined over a longer follow-up period. On the other hand, the risk of tendon rupture was higher in fluoroquinolone-exposed elderly individuals, likely because of the presence of comorbidities that might affect the tendon. Aging might be another factor that affects the pharmacokinetic behavior of antibiotics, relative to the pathophysiological decline of renal function (Pea, 2018).

Routine use of systemic fluoroquinolones should be avoided in children due to the potential risk of joint and musculoskeletal toxicity. However, fluoroquinolones can be used when there are no alternative antibiotics available (Jackson et al., 2016). In Taiwan, the committee for Pediatric Infectious Diseases Society of the Taiwan Child Health Research Center, National Health Research Institutes, and Taiwan Pediatric Association have recommended the use of novel quinolone antibiotics in children since 2012. Systemic use of fluoroquinolones in Taiwan must be administrated by a licensed pediatric infectious disease specialist following an indication, such as macrolide-resistant *mycoplasma pneumoniae* or *Pseudomonas aeruginosa* infection, among others. In this study, we found that patients younger than 18 years exhibited a significantly higher risk of tendon disorder than patients aged 18–59 years. This finding may provide more evidence for clinicians when choosing antibiotics for children.

In the current study, the overall incidence of tendon disorders in patients with and without fluoroquinolone exposure was 6.61 and 3.34 per 10^5^ PYs, respectively. The incidence of tendon disorders was higher among fluoroquinolone-exposed patients with CKD, DM, rheumatologic disease, cardiac disease, and lipid disorders than among those without fluoroquinolone exposure who had these comorbidities. The obesity cases in this cohort were few (five patients with fluoroquinolone exposure and two without), therefore we must be cautious about drawing conclusions. While administering fluoroquinolones to patients with chronic diseases may be necessary, it is crucial to preemptively evaluate the benefits and risks to the patient.

Barring the relationship between fluoroquinolone exposure and tendon disorder occurrence, exposure to statins, aromatase inhibitors, and glucocorticoids is also reportedly related to the occurrence of tendon disorders (Marie et al., 2008; Laroche et al., 2017; Mitsimponas et al., 2018; Wise et al., 2012; van der Linden et al., 2002). In this cohort, we found that only patients who used statins were predisposed to developing tendon disorders. However, the significance of statin use was lost after adjusting for other covariates. Compared with the incidence of tendon disorders in patients who used fluoroquinolones without statins, aromatase inhibitors, and glucocorticoids, the incidence of tendon disorders in patients who used any of these three drug types was more than 10-fold (Supplementary Table S3). Although previous studies showed no relationship between statin use and tendon rupture (Spoendlin et al., 2016; Contractor et al., 2015; Beri et al., 2009), clinicians must be aware of the possible risk of tendon disorders when administering fluoroquinolones to patients taking statins, aromatase inhibitors, or glucocorticoids.

We found that patients with more OPD/ED visits or IPD stay, or a longer ICU stay, may be predisposed to developing tendon disorders, likely due to their more severe disease status. The reason in patients with more OPD/ED visits or IPD stays, or a longer ICU stay, may be a result of the combination of known and unknown comorbidities. Furthermore, those treated in hospitals or ICU may use multiple drug combinations, which may contribute to various drug-drug interactions. This may be one reason for the higher risk of tendon disorders. After comparing patients treated with fluoroquinolones for their OPD/ED visits and IPD/ICU stays, we observed that those treated with IPDs and/or those who had a long ICU stay (indicating a more severe disease status) also showed a higher risk of tendon disorders. Longer durations of fluoroquinolone use (more than 30 days) may also indicate the presence of a more severe infection, which is associated with a relatively higher risk of tendon disorders. This finding corroborated that of a previous study, which demonstrated that the risk of tendon disorder appears to increase by approximately 6% for each day of fluoroquinolone exposure (Morales D. R. et al., 2019). Although the management of patients with more severe infections may require longer durations of fluoroquinolone use, alternative antibiotics may be considered.

We analyzed the risk of tendon disorders associated with the use of seven fluoroquinolones available during the study period: ofloxacin, levofloxacin, ciprofloxacin, norfloxacin, pefloxacin, gemifloxacin, and moxifloxacin. Patients who used levofloxacin, norfloxacin, and pefloxacin had a significantly higher risk of developing tendon disorders. Although the use of the other fluoroquinolones also showed a higher tendon disorder risk, the difference was not statistically significant. We compared the pharmacological or pharmaceutic characters of each fluoroquinolone agent. No correlation between known properties and risk of tendon disorders was identified (Supplementary Table S6). Therefore, more adequate and safe fluoroquinolones should be considered in patients with a higher risk of tendon disorders who need fluoroquinolone treatment. Only a few studies have analyzed the risk of tendon disorders in patients with a combination of comorbidities and medication use. The current study provides more evidence and clarification on the associations among fluoroquinolone exposure, comorbidities, combined medication use, and tendon disorder risk.

### Limitations

Our study has a few limitations. First, the database could not provide actual medical records; hence, we could not thoroughly evaluate whether the symptoms matched the different infection sites for fluoroquinolone use, and therefore, the confirmation of tendon disorders was precluded. Second, the diagnoses in the database were made by different specialists; thus, the diagnoses of tendon disorder may have been determined on different bases. Third, the pathogenesis of tendon disorders is complex and includes contributions from medical history, living environment, and exercise activities that could not be identified in this cohort. Finally, given the retrospective nature of the study, selection bias could not be excluded. In Taiwan, studies on tendon disorders in the general population are limited, and the prevalence of tendon disorders may have been underestimated.

## Conclusion

We have evaluated the risk of tendon disorders in association with fluoroquinolone exposure in Taiwan’s population, using representative population-based data. We have demonstrated that patients with fluoroquinolone exposure were at a significantly higher risk of tendon disorders than the control group. Further studies are therefore needed for patients who need fluoroquinolone treatment, not only on controlling the bacterial infection but also to evaluate the benefits and risks of tendon disorders. We sincerely hope that this study will provide the necessary information to aid clinicians in treating patients with fluoroquinolones.

## Data Availability

The original contributions presented in the study are included in the article/Supplementary Material, further inquiries can be directed to the corresponding author.
