# Supplementary material for: Positive Association Between Fluoroquinolone Exposure and Tendon Disorders: A Nationwide Population-Based Cohort Study in Taiwan
Source: Front Pharmacol. 2022 Mar 21;13:814333. doi: 10.3389/fphar.2022.814333 (PMC8978711; doi:10.3389/fphar.2022.814333)
Supplement: Supplementary file 1 [file DataSheet1.PDF]

Supplementary Material

1 Supplementary Tables

Supplementary Table 1-1. Abbreviation, ICD-9-CM and ICD-10-CM codes and definition

	**Abbreviation**	**ICD-9-CM/Definition**	**ICD-10-CM/Definition**
**Study population:** Fluoroquinolone		Usage ≧3 days; Ofloxacin, Levofloxacin, Ciprofloxacin, Norfloxacin, Pefloxacin, Gemifloxacin, Moxifloxacin	
**Events:** Tendon disorders			
Achilles tendon rupture & tendonitis		726.71, 727.67	M76.60, M66.369
Achilles tendon rupture		727.67	M66.369
Achilles tendonitis		726.71	M76.60
Other tendon diseases		726.10–726.11, 726.19, 726.61, 726.64, 726.72,	M75.50, M75.100, M75.30, M75.80, M76.899, M76.50, M76.829

		727.60–727.66, 727.68-727.69, 727.8-727.9	M66.9, M75.120, M66.829, M66.239, M66.249, M66.339, M66.349, M66.259, M66.269, M66.879, M66.88
**Comorbidities**		≧2 visits; 1 month before the endpoint	
Chronic kidney disease	CKD	585	N18
Diabetes mellitus	DM	250	E10, E11, E13
Obesity		278	E66
Rheumatologic disease		710–719	M05–M25, M30–M36
Cardiac disease		391, 393–398, 402–404, 410–414, 420–429	I00–I02, I05–I09, I11–I13, I20–I25, I30–I5A
Lipid disorders		272	E75, E77, E78, E88,
**Medications**		Usage ≧3 days; 1 month before the endpoint	
Statins			
Aromatase inhibitors			
Glucocorticoid			
**Medical visits**		In the study period	

Outpatient department	OPD	
Emergency room	ER	
Inpatient department	IPD	
Intensive care unit	ICU	

Supplementary Table 1-2. Study drugs, ATC and NHIRD codes

	ATC code	NHIRD code
Fluoroquinolones		
Ofloxacin	J01MA01, S01AE01, S02AA16	AB38213100, AB38554100, AC36508100, AC39523100, AC39793100, AC39797100, AC39798100, AC39819100, AC41542100, AC415421G0, AC42752100, AC42997100, AC44297100
Levofloxacin	J01MA12, S01AE05, A02BD10, J01RA05	AA55545261, AA57191255, AB47516100, AB49696100, AB52615100, AB56626100, AB57191248, AB57839100, AC46634100, AC48694100, AC49452100, AC49505248, AC49505255, AC49651248, AC49651255, AC50104100, AC51715100, AC55545248, AC55545255, AC56750248, AC56750255, AC57101100, AC57185248, AC57185255, AC57220248, AC57220255, AC57220261, AC57242100, AC57300100, AC57376248, AC57376255, AC57376261, AC57822248, AC57822255, AC57822261, AC58044100, AC59017255, AC59831248, AC59888100, BB25828100, BC25068248, BC25068255, BC26772100, BC26837100,

Ciprofloxacin	J01MA02, S01AE03, S02AA15, S03AA07, J01RA10, J01RA11, J01RA12	A038527100, A039271100, A039488100, A039722100, AB58253100, AC36345255, AC36580100, AC37004248, AC37004255, AC37688245, AC38089100, AC38090100, AC38368100, AC38528100, AC38555255, AC38747100, AC39170100, AC40429100, AC41751248, AC41751255, AC44809248, AC44809255, AC44809263, AC45201100, AC47676248, AC47676255, AC47676263, AC47993100, AC49276245, AC49619100, BA18095248, BA18095255, BA18095263, BB17692100, BB21062100, BC24811255, BC26156255
Norfloxacin	J01MA06, S01AE02	A036772100, A037309100, AC29510100, AC33459100, AC33460100, AC33950100, AC39945100, AC40542100, AC41554100, AC415541G0, AC42273100, AC42375100, AC42459100, AC43635100, AC43900100
Pefloxacin	J01MA03	A041639221
Gemifloxacin	J01MA15	AC48275100
Moxifloxacin	J01MA14	A057202265, AC52120265, AC57173100, AC57910100, AC58022265, AC58053265, AC58096265, AC58575265, AC59901265, BC23223100, BC23712265, BC26491100, BC26701100, BC27180265,
Statins	C10AA01-08	AC57216100, AC59251100, AC59887100, BC24250100, BC24391100, BC24392100, BC26169100, BC26582100, BC26643100, BC27283100, BC27534100, BC27535100, A042389100, A043887100, A046022100, A055967100, AA48879100, AA49226100, AA49288100, AA49543100, AA56739100, AA57372100, AA57774100, AA57802100, AA57843100, AA57880100, AA57930100, AA57950100, AA58282100, AA58648100, AB46029100, AB47348100, AB48586100, AB48644100, AB48681100, AB49021100, AB49143100, AB49454100, AB49503100, AB51732100, AB54967100, AB57194100, AB57772100, AB57940100, AB57967100, AB58049100, AC39307100, AC39403100, AC39601100, AC42539100, AC42558100, AC42627100, AC43573100, AC44998100, AC46402100,

		AC47341100, AC47775100, AC47907100, AC47924100, AC47928100, AC48469100, AC48513100, AC48608100, AC48684100, AC48813100, AC48926100, AC49190100, AC49360100, AC49535100, AC49661100, AC49672100, AC49699100, AC49792100, AC49841100, AC49997100, AC50086100, AC51523100, AC51598100, AC52301100, AC52465100, AC52479100, AC52530100, AC52581100, AC55268100, AC55272100, AC55583100, AC55895100, AC55952100, AC55956100, AC56319100, AC56629100, AC56682100, AC56791100, AC56804100, AC56806100, AC57126100, AC57130100, AC57133100, AC57176100, AC57267100, AC57741100, AC57803100, AC57805100, AC57809100, AC58041100, AC58067100, AC58078100, AC58098100, AC58207100, AC58211100, AC58270100, AC58291100, AC58315100, AC58316100, AC58366100, AC58384100, AC58396100, AC58401100, AC58411100, AC58525100, AC58526100, AC58579100, AC58605100, AC58621100, AC58622100, AC58633100, AC58639100, AC58813100, AC58822100, AC59192100, AC59193100, AC59240100, AC59265100, AC59266100, AC59398100, AC59649100, AC59652100, AC60114100, AC60174100, AC60175100, AC60197100, B024297100, B025412100, BA25200100, BA25201100, BA25337100, BA25797100, BA25798100, BA26332100, BA26504100, BC21198100, BC21199100, BC22886100, BC22889100, BC22890100, BC23556100, BC23596100, BC23597100, BC23970100, BC24129100, BC24131100, BC24339100, BC24597100, BC24868100, BC25211100, BC25350100, BC25796100, BC26028100, BC26147100, BC26350100, BC26367100, BC26368100, BC26497100, BC26505100, BC26543100, BC26544100, BC26900100, BC27002100, BC27044100, BC27256100, BC27339100
Aromatase inhibitors	L02BG01-06	AA55555100, AB47539100, AB48909100, AC56730100, AC57732100, AC58819100, BA26108100, BA26206100, BB25535100, BB26515100, BC22282100, BC22462100, BC23097100, BC25143100, BC25251100, BC26127100, BC26313100, BC26564100, BC26581100, BC26641100, BC26791100, BC27015100, BC27448100

Glucocorticoids	H02AB01-17	A001143100, A001226100, A001781100A001833229, A003386209, A003386212, A003386229, A003446221, A003902100, A004073221, A004073299, A004648100, A005185100, A005616229, A006698100, A006749209, A007264100, A007362100, A007681229, A008840100, A008934100, A009300100, A009311100, A0099151G0, A010508221, A010720209, A010720221, A011075209, A011075221, A011075229, A011308209, A012315100, A012488100, A0141841G0, A014692100, A0155791G0, A016065229, A016392100, A016496100, A016879100, A016963221, A017794100, A018669212, A019535100, A019659209, A019659229, A021148100, A022901100, A022941100, A024598100, A024952229, A025420100, A027718206, A027946100, A031274212, A031274229, A033773209, A039045100, A045677100, A055286266, AB13578209, AB13578221, AB13578229, AB14184100, AB14914209, AB14914221, AB14914229, AC00913100, AC01779100, AC02359100, AC02962100, AC03086100, AC030861G0, AC03171100, AC03365100, AC033651G0, AC03494100, AC03534100, AC03700212, AC03700229, AC03881209, AC04158100, AC041581G0, AC04222100, AC04462100, AC04475100, AC04565209, AC04565229, AC05324209, AC05324221, AC06543100, AC06691100, AC07673100, AC07682229, AC08715100, AC08890100, AC08928100, AC09393100, AC09607100, AC096071G0, AC09645209, AC09645221, AC09915100, AC09916100, AC10137100, AC10139100, AC10168100, AC101681G0, AC11125100, AC111251G0, AC11136209, AC11168100, AC11475209, AC11475229, AC11476221, AC11476229, AC11477212, AC11931100, AC11982100, AC12371100, AC12446100, AC12474209, AC12474212, AC12474229, AC12527209, AC12527229, AC13162100, AC13175100, AC13381100, AC133811G0, AC13796100, AC13912209, AC13912229, AC14170100, AC14183100, AC14227100, AC142271G5, AC14325209, AC14325229, AC15268100, AC15279209, AC15279229, AC15467100, AC15579100, AC16065209, AC16065212, AC16464100, AC16589209, AC16963209, AC16963212, AC16963229, AC17256100, AC17292100, AC18670209, AC18670212, AC18670229, AC18679209, AC18946209, AC18946212, AC18946229, AC19418100, AC194181G0, AC19643100,

		AC196431G0, AC19800100, AC20651255, AC20781100, AC21243100, AC21611100, AC21886100, AC21959209, AC21959221, AC21959229, AC22470100, AC22535100, AC23186100, AC23479209, AC23479229, AC23479299, AC23723100, AC24493100, AC24843100, AC24952209, AC24952212, AC25163100, AC26804209, AC26990209, AC26990229, AC27434100, AC27718209, AC29085209, AC29085245, AC29085258, AC29085265, AC29085277, AC29227100, AC29465100, AC29601100, AC296011G0, AC29810100, AC298101G0, AC29931100, AC30311100, AC30695100, AC31676100, AC316761G0, AC31695209, AC31695221, AC31825100, AC318251G0, AC33170100, AC331701G0, AC33184100, AC33187100, AC331871G0, AC33773229, AC33823100, AC338231G0, AC338231G5, AC33894100, AC338941G0, AC34018100, AC340181G0, AC35370100, AC35835100, AC36845100, AC38766100, AC387661G0, AC38955100, AC39305100, AC39740100, AC41020100, AC41029100, AC41358100, AC413581G0, AC41378100, AC413781G0, AC41905100, AC41918100, AC43474100, AC43627100, AC43726209, AC43726245, AC43726265, AC43726277, AC43727100, AC43761100, AC43770100, AC43939100, AC44078100, AC45311100, AC45443245, AC45443258, AC45923100, AC45967100, AC46456100, AC464561G0, AC46461100, AC464611G0, AC464631G0, AC46614135, AC46614143, AC46614151, AC46697100, AC46938100, AC47045100, AC47537245, AC47537258, AC47772100, AC48169100, AC48248277, AC48267245, AC48272100, AC482721G0, AC55920100, AC57313255, AC57385151, AC57749255, AC57811100, AC578111G0, AC57847209, AC57951100, AC57994100, AC58213100, AC58405100, AC584051G0, AC58775100, AC587751G0, AC58821100, BC04248245, BC04922277, BC18167255, N001929100, N002654100, N0045121G0, N004968100, N0052361G0, N005328209, N005328229, N005328299, N005854100, N008799229, N008979100, N009046100, N009059100, N011267229, N011267299, N011556100, N0116071G0, N011760100, N012936100, N013697100, N013702100, NB00220100, NB00711100, NB007111G0, NC00258100, NC00278100, NC002781G0, NC00884100, NC02213100, NC022131G0, NC02531100, NC025311G0, NC02781240,

		NC03097100, NC030971G0, NC04492100, NC04497100, NC04512100, NC05069100, NC05236100, NC06163100, NC06306100, NC063061G0, NC08800209, NC08800229, NC09857100, NC10886209, NC11357209, NC11357229, NC11607100, NC12741100, NC13093100, NC13404100, NC134041G0, NC13481100, NC13906100, NC14296100, NC15101100, NC151011G0, NC15235100, NC16499100

ATC = Anatomical Therapeutic Chemical Classification; NHIRD = National Health Insurance Research Database

Supplementary Table 2-1. Years of follow-up

Fluoroquinolone	Min	Median	Max	Mean±SD
With	0.01	8.87	15.99	10.97±10.86
Without	0.01	8.95	15.99	11.12±10.95
Total	0.01	8.91	15.99	11.05±10.91

Supplementary Table 2-2. Years to tendon disorders

Fluoroquinolone	Min	Median	Max	Mean±SD
With	0.10	6.84	15.23	7.35±7.12
Without	0.89	6.99	15.57	7.69±7.43
Total	0.10	6.90	15.57	7.52±7.28

Supplementary 3. Characteristics of study endpoints

Fluoroquinolone<br>Variables	Total n	Total %	With n	With %	Without n	Without %	*p*
**Total**	714,140		357,070	50.00	357,070	50.00	
**Tendon disorders**							<0.001
Without	713,750	99.95	356,811	99.93	356,939	99.96	
With	390	0.05	259	0.07	131	0.04	
**Sex**							0.999
Male	368,262	51.57	184,131	51.57	184,131	51.57	
Female	345,878	48.43	172,939	48.43	172,939	48.43	
**Age (yrs)**	57.04±19.97		56.93±19.84		57.15±20.09		<0.001
**Age groups (yrs)**							<0.001
0-17	143,576	20.10	72,451	20.29	71,125	19.92	
18–34	192,392	26.94	96,261	26.96	96,131	26.92	
35–59	206,767	28.95	103,433	28.97	103,334	28.94	
≧60	171,405	24.00	84,925	23.78	86,480	24.22	
**Insurance premium (NT$)**							<0.001
<18,000	595,133	83.34	297,121	83.21	298,012	83.46	
18,000–34,999	75,219	10.53	37,608	10.53	37,611	10.53	
≧35,000	43,788	6.13	22,341	6.26	21,447	6.01	
**CKD**							<0.001
Without	649,874	91.00	323,969	90.73	325,905	91.27	
With	64,266	9.00	33,101	9.27	31,165	8.73	
**DM**							<0.001
Without	614,006	85.98	303,925	85.12	310,081	86.84	
With	100,134	14.02	53,145	14.88	46,989	13.16	
**Obesity**							0.779
Without	713,937	99.97	356,966	99.97	356,971	99.97	
With	203	0.03	104	0.03	99	0.03	
**Rheumatologic disease**							<0.001
Without	689,810	96.59	344,619	96.51	345,191	96.67	
With	24,330	3.41	12,451	3.49	11,879	3.33	
**Cardiac disease**							<0.001
Without	647,498	90.67	321,642	90.08	325,856	91.26	
With	66,642	9.33	35,428	9.92	31,214	8.74	
**Lipid disorders**							0.078
Without	707,134	99.02	353,493	99.00	353,641	99.04	
With	7,006	0.98	3,577	1.00	3,429	0.96	
**Statins**							0.010
Without	706,767	98.97	353,274	98.94	353,493	99.00	

With	7,373	1.03	3,796	1.06	3,577	1.00	
**Aromatase inhibitors**							0.101
Without	711,817	99.67	355,869	99.66	355,948	99.69	
With	2,323	0.33	1,201	0.34	1,122	0.31	
**Glucocorticoids**							<0.001
Without	711,263	99.60	355,497	99.56	355,766	99.63	
With	2,877	0.40	1,573	0.44	1,304	0.37	
**OPD/ER visits**							0.002
1–2	77,116	10.80	38,971	10.91	38,145	10.68	
≧3	637,024	89.20	318,099	89.09	318,925	89.32	
**IPD stay (days)**							0.006
0	428,279	59.97	213,478	59.79	214,801	60.16	
1-2	110,717	15.50	55,579	15.57	55,138	15.44	
≧3	175,144	24.53	88,013	24.65	87,131	24.40	
**ICU days**							<0.001
0	538,225	75.37	268,452	75.18	269,773	75.55	
1-6	141,527	19.82	70,505	19.75	71,022	19.89	
≧7	34,388	4.82	18,113	5.07	16,275	4.56	
**Season**							<0.001
Spring	178,136	24.94	89,011	24.93	89,125	24.96	
Summer	180,408	25.26	90,131	25.24	90,277	25.28	
Autumn	178,442	24.99	89,146	24.97	89,296	25.01	
Winter	177,154	24.81	88,782	24.86	88,372	24.75	
**Location**							0.086
Northern Taiwan	200,766	28.11	100,452	28.13	100,314	28.09	
Middle Taiwan	197,890	27.71	98,865	27.69	99,025	27.73	
Southern Taiwan	199,540	27.94	99,973	28.00	99,567	27.88	
Eastern Taiwan	85,757	12.01	42,556	11.92	43,201	12.10	
Outlets islands	30,187	4.23	15,224	4.26	14,963	4.19	
**Urbanization level**							<0.001
1 (The highest)	194,641	27.26	97,305	27.25	97,336	27.26	
2	228,084	31.94	113,298	31.73	114,786	32.15	
3	109,093	15.28	55,972	15.68	53,121	14.88	
4 (The lowest)	182,322	25.53	90,495	25.34	91,827	25.72	
**Level of care**							<0.001
Hospital center	243,096	34.04	121,972	34.16	121,124	33.92	
Regional hospital	248,875	34.85	124,866	34.97	124,009	34.73	
Local hospital	222,169	31.11	110,232	30.87	111,937	31.35	

***p:* Chi-square/Fisher exact test for categorical variables and t-test for continuous variables**

Supplementary Table 4. Sensitivity test for factors of tendon disorders subgroup by using Cox regression with/without Fine–Gray competing risk model

**Sensitivity test**	**Fluoroquinolone**	**With**	**Without** *(Reference)*	**No competing risk in the model**				**Competing risk in the model**			
	**Tendon disorders subgroup**	**Rate (per 10^5 PYs)**	**Rate (per 10^5 PYs)**	**Adjusted HR**	**95% CI**	**95% CI**	***p***	**Adjusted HR**	**95% CI**	**95% CI**	***p***
Overall	Overall	6.61	3.34	1.423	1.023	1.869	0.021	1.449	1.038	1.897	0.011
	Achilles tendon rupture & tendonitis	1.22	0.77	1.152	0.828	1.513	0.172	1.173	0.840	1.535	0.168
	Achilles tendon rupture	0.84	0.51	1.188	0.854	1.560	0.159	1.209	0.866	1.583	0.145
	Achilles tendonitis	0.38	0.26	1.080	0.776	1.418	0.232	1.099	0.788	1.439	0.211
	Other tendon disease	5.38	2.58	1.504	1.081	1.975	<0.001	1.531	1.097	2.004	<0.001
First year excluded	Overall	6.56	3.44	1.372	1.001	1.802	0.049	1.397	1.010	1.829	0.038
	Achilles tendon rupture & tendonitis	1.20	0.78	1.100	0.791	1.445	0.128	1.120	0.803	1.467	0.117
	Achilles tendon rupture	0.82	0.53	1.106	0.795	1.452	0.124	1.126	0.806	1.474	0.109
	Achilles tendonitis	0.38	0.25	1.089	0.783	1.430	0.217	1.109	0.794	1.452	0.205
	Other tendon disease	5.36	2.66	1.452	1.044	1.907	0.005	1.478	1.059	1.936	<0.001
First 5 years excluded	Overall	6.50	3.34	1.399	1.006	1.838	0.045	1.425	1.021	1.866	0.022
	Achilles tendon rupture & tendonitis	0.93	0.52	1.285	0.924	1.688	0.086	1.309	0.938	1.713	0.081
	Achilles tendon rupture	0.67	0.37	1.296	0.931	1.702	0.075	1.319	0.945	1.727	0.073
	Achilles tendonitis	0.26	0.15	1.260	0.905	1.654	0.101	1.283	0.919	1.679	0.094
	Other tendon disease	5.57	2.82	1.421	1.021	1.866	0.023	1.446	1.036	1.894	0.017

PYs = Person-years; Adjusted HR = Adjusted hazard ratio: Adjusted for the variables listed in Table 3.; CI = confidence interval

Supplementary Table 5. Factors of tendon disorders among different models by using Cox regression

Model	Fluoroquinolone	Rate (per 10^5 PYs)	Adjusted HR	95% CI	95% CI	*p*
**Model 0**	Without fluoroquinolone	3.34	Reference			
	With fluoroquinolone	6.61	1.423	1.023	1.869	0.021
**Model 1**	OPD/ER only	6.15	1.323	0.951	1.734	0.097
Patient source	IPD/ICU	7.30	1.572	1.129	2.067	<0.001
**Model 2**	Ofloxacin	6.57	1.412	0.971	1.831	0.083
Fluoroquinolone subtypes	Levofloxacin	6.75	1.456	1.045	1.907	0.018
	Ciprofloxacin	6.64	1.430	0.983	1.784	0.074
	Norfloxacin	6.81	1.468	1.055	1.979	0.005
	Pefloxacin	6.78	1.462	1.048	1.928	0.011
	Gemifloxacin	6.07	1.311	0.935	1.773	0.186
	Moxifloxacin	6.69	1.420	0.996	1.862	0.057
**Model 3**	≦14	6.06	1.302	0.931	1.712	0.201
Fluoroquinolone usage (days)	15–30	6.24	1.345	0.965	1.764	0.098
	≧31	8.32	1.792	1.288	2.359	<0.001

PYs = Person-years; Adjusted HR = Adjusted hazard ratio: Adjusted for the variables listed in Table 3.; CI = confidence interval

Supplementary Table 6. Pharmacokinetics and drug interactions of systemic fluoroquinolones

**Agent**	**Bioavailability**	**Molecular weight (Da)**	**Protein binding**	**Metabolism**	**Excretion**	**Biological half-life (hours)**	**Lipophilicity (Log *p* octanol/water)**
Ofloxacin	>90%	361.4	32%	Glucuronidation	Urine, 65–80% unchanged	9	-0.39
Levofloxacin	99%	361.4	31%	<5% desmethyl and N-oxide metabolites	Urine, 83% unchanged	6–8	2.1
Ciprofloxacin	70%	331.4	30%	Liver	Urine	4.71 (250 mg);	0.28

						3.65 (100 mg)	
Norfloxacin	30–40%	319.3	10–15%	Liver	Urine and fecal	3–4	-1.03
Pefloxacin	100%	333.4	20–30%	Hepatic	Urine, fecal	8.6	0.27
Gemifloxacin	71%	389.4	60–70%	Glucuronidation	Feces (61%); urine (36%)	7	2.3
Moxifloxacin	90%	401.4	47%	38%: Sulphate conjugate; 14% glucuronide conjugate	Urine and feces 45% unchanged drug (~20% in urine and ~25% in feces	11.5–15.6	2.9

The data was from https://pubchem.ncbi.nlm.nih.gov/
